# Can a Continuing Medical Education Intervention Change Pediatricians’ Attention-Deficit/Hyperactivity Disorder Practices?

**DOI:** 10.1016/j.jaacop.2025.10.007

**Published:** 2025-11-07

**Authors:** Peter S. Jensen, Tiffany W. Brandt, Christopher J. Kratochvil, Lisa Hunter Romanelli, James Jaccard

**Affiliations:** a The REACH Institute, New York, New York; b University of Arkansas for Medical Sciences, Little Rock, Arkansas; c Complete Children’s Health Pediatric Center, Lincoln, Nebraska; d University of Nebraska Medical Center and Nebraska Medicine, Omaha, Nebraska; e Silver School of Social Work, New York University, New York, New York

**Keywords:** ADHD, evidence-based practices, continuing medical education, theory of reasoned action, integrated behavior health

## Abstract

**Objective:**

To determine whether a 6-month continuing medical education (CME) program can increase primary care clinicians’ (PCCs) use of attention-deficit/hyperactivity disorder (ADHD) evidence-based practice (EBP) diagnostics and treatment, and to examine whether randomly assigned care manager support further facilitates practice changes.

**Method:**

A total of 47 PCCs attended a 3-day-long CME training, with one-half of PCCs randomly assigned to receive care manager (CM) assistance. All PCCs received continued support via 12 small group teleconference calls over 6 months, After training completion, 9 ADHD EBP variables were abstracted by chart review of 182 newly diagnosed cases seen over a 2-year period (12 months before and after the educational program).

**Results:**

Mixed effects regression analyses examined chart-documented practice changes as a function of pre–post effects of the training and effects of CM assistance, and their interaction. Six of 9 PCCs’ chart-abstracted practice behaviors increased significantly after training, including use of parent–teacher ADHD rating scales at initial diagnosis and over 12 months’ follow-up, as well as side effect monitoring. CM assistance demonstrated additive effects to CME training, but only on 3 of the 9 variables. No training or CM effects were found for 3 other practices: 30-day follow-up visits, total yearly visits, or medication adjustments.

**Conclusion:**

Sufficiently intensive CME programs can produce objective and sustained changes in PCCs’ practices. Additional CM support facilitated some (but not all) of the same changes. Further research is required to determine which practice behavior changes require intensive educational training, CM resources, both, or other practice change interventions.

**Diversity & Inclusion Statement:**

We worked to ensure sex and gender balance in the recruitment of human participants. We worked to ensure race, ethnic, and/or other types of diversity in the recruitment of human participants. We worked to ensure that the study questionnaires were prepared in an inclusive way. One or more of the authors of this paper self-identifies as a member of one or more historically underrepresented racial and/or ethnic groups in science. One or more of the authors of this paper self-identifies as a member of one or more historically underrepresented sexual and/or gender groups in science. One or more of the authors of this paper self-identifies as living with a disability. One or more of the authors of this paper received support from a program designed to increase minority representation in science. We actively worked to promote sex and gender balance in our author group. We actively worked to promote inclusion of historically underrepresented racial and/or ethnic groups in science in our author group. While citing references scientifically relevant for this work, we also actively worked to promote sex and gender balance in our reference list. While citing references scientifically relevant for this work, we also actively worked to promote inclusion of historically underrepresented racial and/or ethnic groups in science in our reference list. The author list of this paper includes contributors from the location and/or community where the research was conducted who participated in the data collection, design, analysis, and/or interpretation of the work.

## Plain language summary

This study examined the impact of an intensive continuing medical education (CME) program on the use of evidence-based diagnostic and treatment practices (EBP) for attention-deficit/hyperactivity disorder (ADHD) among primary care clinicians (N = 47). The CME impact was studied by comparing providers’ charts before and after the CME intervention. Overall, results indicated that this intensive CME intervention increased utilization of EBPs over time, while adding the support of a care manager provided a modest additional benefit on practices.

Evidence-based guidelines for the diagnosis and treatment of attention-deficit/hyperactivity disorder (ADHD) have been available for over 2 decades.^1^, ^2^, ^3^ Despite dissemination efforts, however, substantial gaps remain between the guidelines and their application within real-world practices.^4^, ^5^, ^6^, ^7^, ^8^, ^9^, ^10^ Providers are often faced with obstacles that impede their ability to adhere to guidelines: for example, lack of specific skills, time constraints, insurance restrictions, and difficulties finding educational/psychotherapeutic resources. Short-term continuing medical education (CME) programs often do not address these challenges and obstacles. Perhaps for these and other reasons, critical reviews of typical CME practices (eg, educational meetings) have revealed limited effects on provider practice and behavior change.^11^, ^12^, ^13^

To address these challenges, recent research has examined “behavioral health integration” approaches, that is, strategic combinations of multiple elements, such as the following: (1) care managers (CMs) in behavioral health to conduct patient education, arrange follow-up visits, obtain rating scales, and so forth; (2) on-site counselors to provide brief psychotherapies; (3) psychiatric consultants to advise primary care clinicians (PCCs) or to guide CMs as needed; and (4) computerized registries for patient tracking and progress monitoring. With these combined components, evidence suggests that such interventions can lead to real-world practice changes,^14^, ^15^, ^16^, ^17^—yet, the required resources may not always available. Also, although many adult depression programs have effectively deployed these complex interventions, follow-up studies have shown mixed results as to whether these approaches are universally cost-effective.^17^^,^^18^

For these reasons, alternative approaches should be considered. Strategies to facilitate PCC evidence-based practice (EBP) changes should be reconceptualized as a “behavior change” issue, and the principles and methods from social–psychological behavior change research should be applied to develop more successful PCC practice change efforts.^19^, ^20^, ^21^, ^22^ Strong theoretical and empirical foundations^22^, ^23^, ^24^, ^25^, ^26^, ^27^, ^28^ for behavior change strategies are available, and authors have recently outlined the specific methods and procedures that might be incorporated into PCC EBP training activities to increase their effectiveness.^28^^,^^29^

Consistent with these recommendations, the authors developed a theory-driven practice change intervention designed to facilitate PCCs’ adoption of EBPs for ADHD and other common pediatric mental health conditions (anxiety, depression, etc). The intervention differs substantially from traditional CME programs in several respects. First, it was explicitly designed to optimize PCCs’ beliefs, motivations, and self-efficacy in performing EBPs for optimal mental health disorder outcomes. The intervention and its theoretical basis have been described previously.^30^ Second, consistent with adult learning theory, teachers are carefully selected to be both role models and mentors for practice change, with half of the teacher–coaches consisting of practicing PCCs and the other half child and adolescent psychiatrists. Third, the program assumes that ongoing coaching and problem solving are required, as the PCC invariably encounters obstacles on the way to practice change. The program begins with an initial 3-day hands-on training that includes brief didactics, role modeling, skills practice, and panel discussions. The training is followed by 4 to 6 months of case-based, 1-hour video-conferences in which participants take turns presenting challenging cases from their own practices.

In a previous article examining the program’s impact,^26^ we found that PCCs’ initial and subsequent self-reported use of program-targeted ADHD diagnostic/treatment EBPs increased over time, with these changes mediated by the underlying hypothesized behavioral science predictors: namely, (1) increases in PCCs’ ADHD-related self-efficacy, (2) supportive peer influences, (3) positive beliefs about the new diagnostic/treatment EBPs, and (4) explicit decisions (behavioral intentions) to adopt the new practices.

Surprisingly, however, we found that PCCs who were randomly assigned to receive CM support showed minimal beneficial effects on their subsequent adoption of ADHD EBPs—a finding at odds with common assumptions that CM support is essential in primary care settings. Also of note, PCCs’ initial worries or beliefs concerning practice obstacles decreased significantly over time, again with no apparent impact of CM support. These findings suggested that CME programs—if appropriately designed and sufficiently intensive—can have substantial beneficial impact on PCCs’ EBP behavioral performance, with or without CM support.

**Figure 1:**
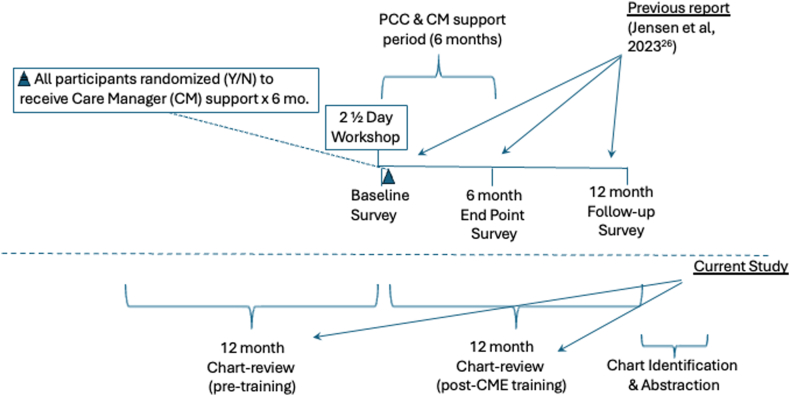
Overall Study Timelines

A major limitation of our first report^26^ is that we did not examine objective measures of PCCs’ actual behaviors, for example, chart reviews. This follow-up report complements our earlier findings by describing the training’s 12-month impact on PCCs’ ADHD diagnostic and treatment EBPs, based on reviews of their patients’ charts. Furthermore, to cross-check the validity of PCCs’ previously reported practice change intentions,^26^ we examine whether their change intentions after the CME (eg, to use ADHD rating scales) are correlated with their subsequent practices as revealed in their patients’ charts. As a final step, here we also compare PCCs’ ADHD chart-documented practices 1 year before the actual workshop vs chart-documented practices for 1 year after the workshop, thereby allowing PCCs to serve as their own historical controls. Figure 1 provides an overall description of the study design and how the first report relates to these follow-up analyses.

## Method

The aims of this report are as follows: (1) to present objective data concerning the impact of an intensive CME program on PCCs’ ADHD diagnostic and treatment practices, based on chart reviews of patients newly diagnosed with ADHD over 12 months before training and 12 months immediately after the initial workshop; (2) to examine the impact of randomly assigned CMs to assist PCCs with implementing the study-targeted diagnostic and treatment EBPs; and (3) to determine the correlations between PCCs’ previously stated behavioral intentions (described in our first report^26^) to change their ADHD practices vs their actual chart-documented practices (this report).

### Setting and Participants

Study participants were recruited from the AccessCare network, a private non-profit 501 (c)(3) primary care regional (24-county) provider network the mission of which is to improve quality and cost-effective care for Medicaid recipients in the state of North Carolina. The network consists of a large number of physician-owned primary care practices, with approximately 50 child/adolescent-focused primary care clinics. AccessCare serves over 27% of the state’s pediatric Medicaid population, with participating practices focused on quality improvement initiatives and connecting Medicaid enrollees with a medical home. In all, 23 of 24 of the network’s covered counties are rural. Given its long-standing focus on improving care quality, network leaders readily agreed to participate in this initiative, framed as a quality improvement study.

Pediatric practices were requested to identify 1 participant per practice, to avoid within-practice clustering effects. Because only 30 practices volunteered, the original objective of 1 participant per practice was relaxed, and practices were allowed to enroll additional participants per practice (power analyses indicated that 60 participants were likely needed). A total of 47 PCCs agreed to participate in the initial study, but only 43 PCCs agreed to allow review of their patient records Of these, 42 PCCs provided demographic information indicating that the majority (27) identified as female (64.3%) and had an average age of 48.0 years (SD = 9.67). For professional discipline, 30 (71.4%) PCCs trained as pediatricians, 4 (9.5%) trained in family medicine, and 7 (16.7%) trained as non-physicians (physician assistants or nurse practitioners, all with prescribing privileges). Participating PCCs saw an average of 95.0 (SD = 48.6) patients per week. PCCs averaged over 3113 Medicaid-enrolled children in their practices, based on Medicaid claims data in the year before the study, with 27% and 17% of these children identified as African American and Latino, respectively.

### PCC Training Procedures

All PCCs agreed to attend an initial 3-day training, the Patient-Centered Mental Health in Pediatric Primary care Program (PPP), a CME training program^30^ developed by the non-profit REACH Institute (www.TheReachInstitute.org). The main goal of training was to encourage PCCs to implement EBPs for diagnosing and treating pediatric mental health problems such as depression, anxiety, ADHD, and conduct disorders. The training consisted of didactic instruction, live simulations of patient/family interviews, table work, hands-on practice in new skills, and question-and-answer panels. Participants were provided multiple resources to enhance their clinical practices, including medication dosage charts, rating scales, patient handouts, and so forth. Although the PPP training addressed all common pediatric mental health conditions (depression, anxiety, oppositional defiant disorder, etc), for the purposes of this study, only PCC behaviors specific to ADHD were abstracted. After the initial 3-day training, PCCs were encouraged to attend 12 follow-up consultation support calls in small conference call groups for 6 months. Actual call attendance averaged 7.4 calls (SD = 4.3, range 0-12). PCCs were reimbursed $150 for completion of each of 3 surveys (as described in our first report): after the workshop, after the 6 months’ support calls, and at 12 months post workshop.

### Care Manager Procedures

At the end of the 3-day PCC training, PCCs were randomized to receive or not to receive the additional support of a CM. CMs were already employed within the AccessCare system, but were originally focused on assisting PCCs in providing essential elements of the medical home, with no responsibilities for ADHD or other behavioral health diagnoses. Usual CM duties included care coordination, developing individualized action plans for patients with chronic illnesses (eg, asthma action plans), connecting families with social services when needed, and addressing social determinants of health, particularly where such factors might affect difficulties accessing and follow-up with pediatric services. For the purposes of this study, AccessCare administrators agreed to reassign care managers (by random assignment at the practice level) to specifically assist PCCs to improve children’s ADHD care.

Consistent with their previous responsibilities, CMs’ ADHD-related duties were as follows: (1) to meet with individual families to discuss the ADHD diagnosis and to provide additional education and support handouts; (2) to assist PCCs in obtaining parent and teacher ADHD rating scales; (3) to follow up with families who might miss appointments; (4) to meet with families in their homes (if requested and as needed) for further follow-up and discussion about the diagnosis and its treatment; (5) to facilitate mental health referrals if needed or requested; and (6) to communicate relevant information to PCCs about family concerns with the diagnosis and/or treatment.

To assist CMs in learning these new duties, they participated in a separate 1-day training concerning the following: 1) the ADHD diagnosis and assessment processes; 2) use, benefits, and side effects of medications; 3) use, scoring, and interpretation of parent–teacher ADHD rating scales; 4) the role and importance of parent behavioral training; (5) strategies for encouraging medication adherence; and (6) methods for helping parents obtain additional school supports (504 plans, individualized education programs [IEPs]).

After their initial training (completed at the same time as the PCCs’ initial workshop), CMs participated for 6 months in twice-monthly, 1-hour support/problem-solving teleconference sessions with 1 of the study investigators (PSJ), an AccessCare leader, and an experienced CM supervisor. From that point forward, CMs assisted their assigned PCCs in the following: patient/family ADHD individualized education, meeting each newly diagnosed patient’s caregiver (after receiving notification from the PCC) to explain the diagnosis, addressing family questions/concerns, assisting with collection of parent–teacher ADHD rating scales, offering assistance to facilitate school supports, and helping the family make/complete any recommended/requested therapy referrals. CMs supported an average of 2 ADHD patients (range 1-3), made 3.1 patient/family contacts per month, and averaged 1.25 hours per month assisting these patients/families.

To enhance CMs’ ADHD-related family support skills and to enhance overall program quality, supervisors facilitated CMs’ regular support call attendance, offered additional assistance as needed, and followed up individually with any CMs missing the regular support calls. Formal attendance data were not maintained, but call facilitators estimated that CMs averaged 70% for overall attendance. It should be noted that the 6-month phone support periods for both the PCCs and the CMs ran concurrently (Figure 1). CMs received $100 for attending their initial day-long ADHD training.

### Measures

Investigators identified 9 PCC practice behaviors that were explicitly taught or encouraged during the training and that also could be abstracted or counted from each PCC’s ADHD case records: (1) obtaining ADHD rating scales from both parent and teacher informants (to help establish the initial diagnosis); (2) when prescribing medication, to titrate it upward from an initial low dose to a maximally effective dose; (3) to conduct follow-up visits within 30 days if a medication was started (a Healthcare Effectiveness & Data Information Set [HEDIS] quality metric^31^^,^^32^); and (4) to identify, track, and manage initial side effects. For the second variable, “to titrate upward from an initial low dose….”, ascertaining an “effective maximal dose” could not be easily be performed by chart review, so this chart screening variable was changed to simply ascertaining whether the clinician prescribed any higher dose during the first 3 months after stimulant initiation. These 4 variables were collected as simple counts (eg, multiple teachers might fill out an initial ADHD rating scale, yielding a numeric sum >1), and could also be examined as binary (yes/no) EBP quality indicators. Similarly, a family might return for more than 1 follow-up visit within 30 days after an initial medication prescription.

For EBPs related to longer-term ADHD management, PCCs were encouraged to monitor patients with frequent (2 or more) follow-up visits within the next 270 days after the first follow-up (also a HEDIS metric). Accordingly, the number of follow-up visits were counted; for recording convenience, the follow-up chart abstraction period was extended to 12 months post workshop. Three additional long-term management data points were gathered: counts of parent and teacher ADHD rating scale chart mentions (a presumptive “best practice” emphasized during training to track treatment response^33^); and counts/mentions of PCCs’ assessing/addressing treatment-emergent side effects (eg, sleep, appetite, weight, stomach/headaches, or other concerns) (Table 1, four corresponding rows under “Follow-up Treatment and Management Period [90-365 days]”). This table separately reports these data for both 1 full year before the CME workshop and 1 full year thereafter.

**Table 1:** Mean Counts/Mentions Per Patient Chart (Total Charts = 182)

Chart variable	Mentions/chart	Range	SE
**Pre training (12 mo before CME workshop) (n = 90)**			
Initial diagnostic and treatment period (up to 90 days)			
Initial parent ADHD scale	0.71	0-2	0.06
Initial teacher ADHD scale	0.70	0-3	0.06
Initial medication adjustment	0.39	0-3	0.07
Initial side effects management	0.98	0-4	0.10
F/U ≤30 days of prescription	0.83	0-3	0.07
Follow-up treatment and management period (90-365 days)			
Total parent ADHD F/U scales	0.84	0-5	0.09
Total teacher ADHD F/U scales	0.79	0-4	0.07
Total side effects mentions	1.88	0-8	0.19
Total F/U Visits	3.86	0-14	0.30
**Post training (12 mo immediately after CME workshop) (n = 92)**			
Initial diagnostic and treatment period (up to 90 days)			
Initial parent ADHD scale	1.10	0-3	0.06
Initial teacher ADHD scale	1.02	0-3	0.07
Initial medication adjustment	0.53	0-4	0.09
Initial side effects management	1.41	0-8	0.13
F/U ≤30 days of prescription	0.99	0-3	0.08
Follow-up treatment and management period (90-365 days)			
Total parent ADHD F/U scales	1.47	0-5	0.11
Total teacher ADHD F/U scales	1.42	0-5	0.12
Total side effects mentions	2.76	0-11	0.27
Total F/U visits	4.41	0-14	0.32

Note: All numbers in the “Mentions/Chart” column are average counts of the number of instances (mentions) that a given behavior/practice was found across all patient records in that time period (pre or post training). For example, for the first variable, “Initial Parent ADHD Scale,” an average of 0.71 ADHD rating scale was noted per patient chart, ie, <1. ADHD = attention-deficit/hyperactivity disorder; F/U = follow-up.

### Chart Selection

After the completion of PCCs’ final 12-month surveys (see previous report^26^), retrospective records reviews were performed to identify all patients with newly diagnosed ADHD within either of two 12-month planned comparison periods: 12 months before the workshop or 12 months after the workshop (Figure 1). A total of 182 charts of children newly diagnosed within the 2 chart assessment periods were identified, 90 charts in the 12 months prior vs 92 charts in the 12 months after the initial workshop, averaging 4.3 patient records per PCC. Because all chart cases had to be newly diagnosed within the two 12-month chart review periods, chart case length was variable, based on whenever PCCs diagnosed and charted the cases. Thus, across both charting periods, case follow-up length (from time of diagnosis to/from the workshop date) averaged 7.35 months, roughly comparable between the pre- and post-workshop chart review periods (7.2 vs 7.5 months, respectively). Given the importance of these data and the variable charting periods, each patient’s charting period length was included in all analyses, as described below.

### Data Analysis

Given the expected non-normal distributions of simple count data, normality was assessed for all outcome variables using the Shapiro–Wilk test, histogram visualization, and skewness measures. All variables showed moderate to high right skew (skewness >1) and were log-transformed to improve distributional assumptions before analyses.

PCC practice behaviors (outcomes in Table 1) were analyzed using mixed-effects repeated-measures models. Both binary (yes/no) and ordinal/continuous variables were included. PPC identity (ID) and practice ID were modeled as random intercepts to account for clustering, with fixed effects for time (baseline vs follow-up), CM (yes/no) assignment, their interaction, number of support calls attended, and each chart’s individual follow-up length.

Because PCCs (n = 43) were nested within practices (n = 30, mean cluster ∼1.5 PCCs), we tested for practice-level clustering. Variance components were small or near zero, indicating little practice-level effect once individual PCC variation was modeled. To avoid overfitting, only random intercepts were retained; random slopes for practice by time or CM did not improve model fit.

Initial 90-day outcomes were coded 0/1 and analyzed as linear probability mixed models. Event rates were moderate (30%-80%) and fitted values stayed within 0 to 1, making this approach appropriate. Longer-term (90-365 days) outcomes were treated as continuous. Log(x+1) transformations were explored but yielded the same results, with distributions reasonably symmetric. Significant Time × CM interactions were interpreted as evidence of additive CM impact, and main effects of CM were not expected. A missingness indicator was included in all models.

### Cross-Validation of Chart Measures With PCCs’ Stated Practice Change Intentions

To cross-check the consistency of the chart-abstracted measures with PCCs’ explicitly stated intentions to change specific ADHD practices (described in our first report), we examined the simple correlations between the log-transformed chart data and PCCs’ previously assessed corresponding behavioral intentions to do the following: (1) use parent/teacher rating scales for initial assessments, (2) use rating scales to monitor long-term treatment monitoring, (3) conduct frequent follow-up visits, and (4) track/measure side effects. All behavioral intentions (BIs) were scaled from 0 (I do not intend to perform this behavior…) to 10 (I strongly intend to perform this behavior…).

## Results

As shown in Table 1, for all outcomes, the average number of counts/mentions per patient chart are frequently less than 1.0, but the range as high as 13, indicating the need for log(x+1) transformations. Because many of these values are computed on the basis of 1 patient per chart, table inspection indicates relatively high proportions of patients with initial/diagnostic parent and teacher ADHD scales (0.70, 0.71, respectively). Similarly, most patients’ charts reflected the presence of medication follow-up visits within 30 days (0.83), as well as high average numbers of 12-month follow-up visits after the initial first medication follow-up visit (2.86), both findings consistent with HEDIS guidelines.^32^

Table 2 reveals the key variables abstracted during the initial diagnostic/treatment period, within the first 3 months. This timeframe was set to accommodate practice variations in collecting both parent and teacher ratings scales during the initial diagnostic assessment. Of the five tabled variables, 3 showed a significant pre–post time/training effect, that is, with increases in parent/teacher rating scale counts and side effects mentions. One significant CM additive interaction was noted: teacher rating scale documentation. No increased mentions were found in performing ≤30-day medication follow-up visits, perhaps a result of the already high proportion of charts meeting that criterion during the pre-workshop period (0.79-0.91 mentions/chart).

**Table 2:** Impact of Training and Care Manager Status on Initial Diagnosis and Treatment

Outcome variable (chart mentions)	Pre vs post training			Care manager status (yes/no)			Interaction			*R*^*2*^ (adj)	Average no. of mentions/chart (SE)		
	*F*	*df/ df*_*Density*_	*p*	*F*	*df/ df*_*Density*_	*p*	*F*	*df/ df*_*Density*_	*p*		CM assignment	Pre training (n = 90)	Post training (n = 92)
No. of parent ADHD scales at initial diagnosis	19.7	1, 34.9	≤.0001^a^	2.1	1, 36.9	≤.16	3.3	1, 34.9	≤.09	0.48			
											No CM	0.84 (0.09)	1.08 (0.10)
											CM	0.55 (0.10)	1.11 (0.11)
No. of teacher ADHD scales at initial diagnosis	14.3	1, 33.7	≤.0006^a^	0.2	1, 37.0	≤.70	7.8	1, 33.7	≤.01^a^	0.48			
											No CM	0.76 (0.11)	0.85 (0.11)
											CM	0.71 (0.12)	1.24 (0.12)
No. followed up within 30 days of prescribing	1.6	1, 28.9	≤.22	0.0	1, 28.0	≤.96	0.0	1, 28.9	≤.96	0.17			
											No CM	0.79 (0.11)	0.95 (0.11)
											CM	0.91 (0.12)	1.02 (0.12)
No. with initial side effects monitoring	5.9	1, 36.1	≤.02^a^	3.6	1, 31.4	≤.07	0.6	1, 36.1	≤.44	0.38			
											No CM	0.83 (0.18)	1.11 (0.18)
											CM	1.11 (0.20)	1.72 (0.19)
Initial medication adjustment and titration	1.2	1, 31.7	≤.27	0.9	1, 29.0	≤.34	2.6	1, 31.7	≤.12	0.52			
											No CM	0.40 (0.14)	0.33 (0.14)
											CM	0.38 (0.15)	0.76 (0.15)

Note: ADHD = attention-deficit/hyperactivity disorder; adj = adjusted; CM = care manager.

a Statistically significant.

Table 2 also presents the case-based counts of medication changes made by PCCs during the first 3 months post diagnosis, revealing no differences in medication changes, either by pre–post period or by CM status (pre/CM+: 0.38 changes/case; post/CM+: 0.76; pre/CM−: 0.40; post/CM−: 0.33). Given the relative infrequency and variability of medication changes per case, much larger samples of patients and PCCs would likely be needed to determine effects on this clinical behavior. In these mixed-effects models, variance attributable to practice membership was minimal. Practice-level random effects were small or estimated as zero, indicating that outcomes did not cluster meaningfully within practices. Accordingly, the key results are interpreted at the physician level, with practice effects treated as negligible. Results were unchanged when practice effects were modeled as random slopes.

**Figure 2:**
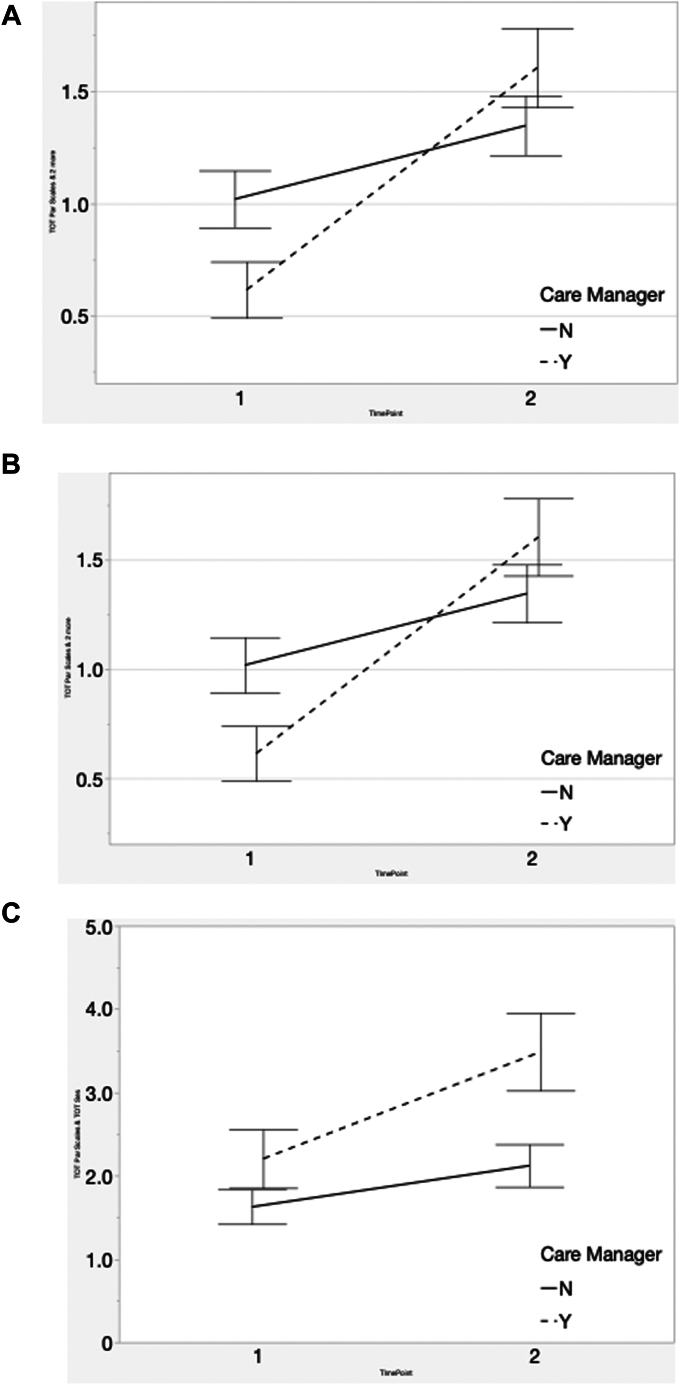
Effects of Care Manager Support, 12-Month (Pre–Post) Periods ***Note:** (A) mean total parent adhd scales per chart, by care manager. (B) Mean total teacher attention-deficit/hyperactivity disorder (ADHD) rating scales per chart, by care manager. (C) Mean total side effects mentions per chart, by care manager.*

Table 3 presents the treatment/management follow-up period variables that were abstracted for the remainder of the 12 months—all behaviors emphasized during training. The parent/teacher rating scale findings are notable, illustrating the apparent additive effect of CM assistance on pre–post training impact. For side effects mentions, a pre–post training effect was again found, but without CM additive affect. Table 3 also reveals the absence of either pre–post training or CM effects on the number of follow-up visits by 12 months, maybe because of the high number of follow-up visits reported even before training, possibly a “ceiling” effect. To illustrate these 3 significant Table 3 findings, Figure 2 presents these data visually; the additive effects of CM status on training impact are easily seen.

**Table 3:** Impact of Training and Care Manager Status on 12-Month Management

Outcome variable	Pre vs post training			Care manager status (yes/no)			Interaction			*R*^*2*^ (adj)		Average no. of mentions/chart	
	*F*	*df/ df*_*Density*_	*p*	*F*	*df/ df*_*Density*_	*p*	*F*	*df/ df*_*Density*_	*p*		CM assignment	Pre training n = 90 (SE)	Post training n = 92 (SE)
No. of parent ADHD scales, 12 mo	21.4	1, 36.6	≤.000^a^	1.1	1, 40.0	≤.30	5.2	1, 36.6	≤.03^a^	0.48			
											No CM	1.01 (0.16)	1.34 (0.16)
											CM	0.61 (0.17)	1.58 (0.17)
No. of teacher ADHD scales, 12 mo	21.9	1, 33.0	≤.000^a^	0.5	1, 37.5	≤.47	7.5	1, 33.0	≤.01^a^	0.53			
											No CM	0.83 (0.17)	1.13 (0.17)
											CM	0.81 (0.18)	1.75 (0.18)
No. of follow-up visits, 12 mo	1.3	1, 30.5	≤.26	1.3	1, 36.4	≤.26	1.0	1, 30.5	≤.32	0.32			
											No CM	3.22 (0.50)	4.06 (0.51)
											CM	4.79 (0.54)	4.69 (0.52)
No. of side effect mentions, 12 mo	5.0	1, 34.4	≤.04^a^	2.1	1, 37.1	≤.16	0.6	1, 34.4	≤.44	0.48			
											No CM	1.65 (0.40)	2.10 (0.41)
											CM	2.19 (0.43)	3.48 (0.42)

Note: ADHD = attention-deficit/hyperactivity disorder; adj = adjusted; CM = care manager.

a Statistically significant.

To examine the correlations between PCCs’ earlier stated behavioral intentions (BIs) vs their actual practices, Table 4 presents the simple correlations between BI strength (possible range 0-10) and the chart-documented counts for behaviors that increased significantly in Tables 2 and 3; that is, initial use of parent/teacher rating scales and side effects mentions (Table 2), and the same 3 variables over 12 months (Table 3).

**Table 4:** Correlations Between Chart Counts and Primary Care Clinicians’ Declared Practice Change Intentions Regarding Use of Parent and Teacher Attention-Deficit/Hyperactivity Disorder Rating Scales

Behavioral intention	Chart abstraction period	Pearson *R*	Significance, *p*	Total n	*R*^*2*^ (adj)
To obtain initial parent scales	Post to 3 mo	0.45	≤.009^a^	33	0.18
To obtain initial teacher scales	Post to 3 mo	0.45	≤.008^a^	33	0.18
To assess initial side effects	Post to 3 mo	0.04	≤.84	34	−0.03
To obtain ongoing parent scales	Post to 12 mo	0.38	≤.04^a^	31	0.12
To obtain ongoing teacher scales	Post to 12 mo	0.35	≤.06	31	0.09
To monitor ongoing side effects	Post to 12 mo	0.38	*≤*.03^a^	33	0.12

Note: All behavioral intention variables reported here were derived from the first of the 3 assessment surveys described in our previous study.^26^ This caveat is important, because behavioral intentions can wane or change over time for various reasons, for example, if performing the new behavior is more difficult than expected, if it does not yield expected benefits, if one subsequently encounters obstacles to behavioral performance, or if one learns of a more preferable alternative. adj = adjusted.

a Statistically significant.

## Discussion

The first report from this study^26^ indicated that a theory-driven CME training for PCCs yielded both immediate and sustained (12-month) behavioral intentions/commitments to apply ADHD diagnostic and treatment EBPs. This report extends these findings by demonstrating the intervention-generated objective/chart-documented practice changes for 6 of 9 targeted practices. These findings are noteworthy, in view of evidence that CME programs often fail to produce changes in clinicians’ day-to-day practices, rarely over 12 months.^11^^,^^12^ Surprisingly, but also of note, we found that additional CM support showed benefits on only 3 of 9 variables, with little to no effect on any other chart-abstracted behaviors.

Why should this be the case, and when might an intensive longitudinal CME intervention focused on motivating and supporting optimal PCC practices be either sufficient by itself or considered as an essential element of an overall practice transformation program? When might the additional resources of CMs be required over and above an intensive CME program, and what are the relative costs/benefits of either or both components? Relatedly, when should health care planners add provisions for co-located psychotherapeutic resources, psychiatric consultants, tracking registries, or other elements outlined in many integrated behavioral health care models?^34^ Although many studies have documented the impact and likely cost-effectiveness of combined elements within integrated behavioral health care programs in adult primary care,^35^ few studies have been conducted within pediatric primary care settings,^36^ fewer still with ADHD specifically, and in the current literature, none explicitly detailing the relative costs/benefits of each of these components.^34^

Moreover, although several broad-scale pediatric integrated behavioral health care interventions have demonstrated their feasibility (usually limited to university-affiliated, single-payor, or geographically constrained settings),^15^^,^^37^^,^^38^ such efforts may be difficult to scale more broadly or directly transfer to non-academic, private practice, or rural settings. Although programs to date offer the hope that such systemic reorganization efforts might ultimately be proved to be cost-effective, to date only 1 pediatric study has provided formal cost–benefit analyses, albeit in a small sample of Australian children with non-complex medical conditions (58% of the sample with ADHD).^39^

Thus, most available studies do not disaggregate the various elements of integrated behavioral health care models, such that one can determine which elements are crucial for effective primary care mental health services, or which are most essential from an economic or cost–benefit perspective.^36^ Although some innovative Web-based ADHD assessment and treatment tracking systems have been developed,^40^ such methods may not invariably improve PCCs’ practices, even when coupled with several hours of CME training.^41^

Given the challenges, costs, and complexity of reorganizing pediatric primary care settings to deliver mental health services, the merits of an easily scalable and transferable CME program seem easily justifiable if the program can reliably produce PCC EBP changes (as found here) when large-scale reorganization resources are not available. In this regard, several studies have found that this intervention may yield cost savings within integrated, single-payer health care systems or even within a large regional Medicaid provider network.^42^, ^43^, ^44^

Despite the promising findings in this report, limitations should be considered. First, the number of patient charts per clinician was modest, and it is quite possible that more robust CM effects might be found with more cases, better trained CMs, or longer follow-up periods. In addition, important clinical practices, for example, PCCs’ titrating/adjusting medication doses based on rating scales or interview elements, may not be easily gleaned from chart reviews. Other behaviors might be relatively infrequent or inconsistently documented, for example, making mental health referrals when psychotherapeutic resources are outside of the medical records system. Other clinical practices (eg, changing to a non-stimulant if 2 stimulant classes have failed) may occur only after 1 to 2 years of treatment efforts. Longer-during studies might be required to more carefully study such practices, and might require in-depth interviews with PCCs to better understand the nature and rationale of what otherwise might appear to be an obscure practice found in the medical record.

Moreover, it should be noted that all participating PCCs had joined this particular Medicaid network with its well-known strong focus on practice improvement, and then further volunteered to participate in this improvement activity. It is unclear whether these activities and findings would apply to less motivated, less prepared PCCs.

In addition, some authors have noted that PCCs’ reports of specific ADHD EBPs may not correlate reliably with chart data.^45^ This is an important problem, but we found here that PCCs’ stated behavioral intentions to perform future behaviors are in fact linked, albeit imperfectly, to some (but not all) of their subsequent practices (Table 4). In fact, multiple studies have shown that behavioral intentions are the single best predictor of future behavior performance, particularly for behaviors that follow closely in time explicitly stated behavioral intentions.^21^^,^^46^

A final limitation should be noted: no controlled data were available, that is, assessing changes in chart measures drawn from untrained PCCs during the same time frames. This limitation might mean that the pre–post charting changes were due to simple time effects rather than to the training program itself. Counterbalancing that concern is our finding that PCCs’ stated behavioral intentions to use rating scales and to conduct side effect assessments correlated robustly with actual chart measures in 4 of 6 measures (Table 4).

This study builds upon previous research documenting the benefits of a 6-month–long, theory-driven CME intervention on PCCs’ ADHD-related EBPs, with some effects augmented by CM support. However, our findings also revealed instances where these interventions, singly or combined, had no discernible effects on PCCs’ practices. To further improve the health care quality for ADHD and other pediatric mental health conditions, future studies should examine larger and more representative PCC samples to determine the single vs combined effects of intensive vs less intensive CME trainings and CM support, and compare and contrast these strategies with other practice change elements, such as embedded psychotherapy resources, automated reminders and feedback systems, and clinical registries.

## CRediT authorship contribution statement

**Peter S. Jensen:** Writing – review & editing, Writing – original draft, Supervision, Project administration, Methodology, Funding acquisition, Formal analysis, Conceptualization. **Tiffany W. Brandt:** Writing – review & editing, Writing – original draft, Formal analysis. **Christopher J. Kratochvil:** Writing – review & editing, Methodology, Investigation. **Lisa Hunter Romanelli:** Writing – review & editing, Supervision, Project administration, Funding acquisition, Conceptualization. **James Jaccard:** Writing – review & editing, Formal analysis.
